# Developing transcriptomic biomarkers for TAVO412 utilizing next generation sequencing analyses of preclinical tumor models

**DOI:** 10.3389/fimmu.2025.1505868

**Published:** 2025-02-10

**Authors:** Ying Jin, Peng Chen, Huajun Zhou, Guangmao Mu, Simin Wu, Zhengxia Zha, Bin Ma, Chao Han, Mark L. Chiu

**Affiliations:** ^1^ Research & Development Department, Tavotek Biotherapeutics, Suzhou, Jiangsu, China; ^2^ Global Center for Data Science and Bioinformatics, Crown Bioscience Inc., Suzhou, Jiangsu, China; ^3^ Research & Development, Tavotek Biotherapeutics, Spring House, PA, United States

**Keywords:** EGFR cancer cells +, cmet, VEGF - vascular endothelial growth factor, antibodies, PDX (patient derived xenograft), CDx, transcriptome

## Abstract

**Introduction:**

TAVO412, a multi-specific antibody targeting epidermal growth factor receptor (EGFR), mesenchymal epithelial transition factor (c-Met), and vascular endothelial growth factor A (VEGF-A), is undergoing clinical development for the treatment of solid tumors. TAVO412 has multiple mechanisms of action for tumor growth inhibition that include shutting down the EGFR, c-Met, and VEGF signaling pathways, having enhanced Fc effector functions, addressing drug resistance that can be mediated by the crosstalk amongst these three targets, as well as inhibiting angiogenesis. TAVO412 demonstrated strong *in vivo* tumor growth inhibition in 23 cell-line derived xenograft (CDX) models representing diverse cancer types, as well as in 9 patient-derived xenograft (PDX) lung tumor models.

**Methods:**

Using preclinical CDX data, we established transcriptomic biomarkers based on gene expression profiles that were correlated with anti-tumor response or distinguished between responders and non-responders. Together with specific driver mutation that associated with efficacy and the targets of TAVO412, a set of 21-gene biomarker was identified to predict the efficacy. A biomarker predictor was formulated based on the Linear Prediction Score (LPS) to estimate the probability of patients or tumor model response to TAVO412 treatment.

**Results:**

This efficacy predictor for TAVO412 demonstrated 78% accuracy in the CDX training models. The biomarker model was further validated in the PDX data set and resulted in comparable accuracy.

**Conclusions:**

In implementing precision medicine by leveraging preclinical model data, a predictive transcriptomic biomarker empowered by next-generation sequencing was identified that could optimize the selection of patients that may benefit most from TAVO412 treatment.

## Introduction

TAVO412 was a multiple specific antibody targeting epidermal growth factor receptor (EGFR), mesenchymal epithelial transition factor (c-Met), and vascular endothelial growth factor (VEGF-A). In addition to the multiple target specificities, this antibody was engineered to have enhanced effector functions such as antibody-dependent cell-mediated cytotoxicity (ADCC), antibody-dependent cell-mediated phagocytosis (ADCP), and complement-dependent cytotoxicity (CDC) to potentiate its antitumor effects (1).

Dysregulated and/or mutated EGFR and c-Met lead to tumor cell survival, proliferation, invasion, migration, and development of drug resistance. Solid tumor growth and migration also involves angiogenesis, where VEGF-A plays a key role in expanding tumor neovasculature, aiding tumor growth and metastasis. VEGF-A activates angiogenesis through the vascular endothelial growth factor receptor 2 (VEGFR2) in an autocrine and paracrine manner (2). The crosstalk among EGFR, c-Met, and VEGF signal pathways are one of the major mechanisms of drug resistance (3–5). Simultaneous shutting down EGFR, c-Met and VEGF signaling showed promising results in preclinical animal models (6). TAVO412 has demonstrated very promising antitumor activities in animal models including non-small cell lung cancer (NSCLC), gastric cancer (GC), pancreatic ductal adenocarcinoma (PDAC), and triple negative breast cancer (TNBC) (1). In addition, TAVO412 also demonstrated activities in tumor types with dysregulated EGFR, c-Met, and VEGF signaling, such as head and neck squamous cell carcinoma (HNSCC) (7–9), ovarian cancer (OC) (10), hepatocellular carcinoma (HCC) (11), and small cell lung cancer (SCLC) (12). Moreover, TAVO412 demonstrated efficacies in NSCLC and SCLC PDX models with stronger activities than amivantamab, an EGFR x c-Met bispecific antibody recently approved for EGFR Exon 20 insertion–driven NSCLC (13). Such a rich data set of TAVO412 in multiple tumor types in the preclinical models allows further explorations of potential predictive biomarkers.

The responses to antitumor treatment can vary significantly among individual patients (14), even with identified tumor-driving mechanisms and targeted-therapies, due to the heterogeneity of tumor cells and the tumor microenvironment, as well as the complex interactions between the tumor and its surroundings. Developing predictive biomarkers for patient selection is crucial for a successful clinical development. Empowered by the recent development of next-generation sequencing, transcriptome profiling emerged as a powerful approach in oncology for the predictive utilities in specific disease management. Differentiating from genomic biomarkers, transcriptomic approaches can reflect the dynamic status of tumor growth and its biologic environment by presenting holistic information of gene expressions while providing much improved continuity and ease in the analysis compared to proteomics (15). Transcriptomic biomarkers can provide a comprehensive benchmark for immune checkpoint agents in the clinic (16). Examples of developing predictive biomarkers using preclinical tumor models also have been published (17).

During the preclinical development of TAVO412, the antitumor effects were evaluated in various tumor cell line-derived xenograft (CDX) models as well as in patient-derived tumor (PDX) models with different EGFR and c-Met expression levels and mutations. While TAVO412 demonstrated significant tumor inhibition in most models, target expression alone was not sufficient to explain the variability in the responses. To develop predictive transcriptomic biomarkers, correlations between gene expressions and efficacy responses were extensively analyzed. Based on the RNA sequencing data of the CDX tumor tissues, a comprehensive gene expression profiling identified 19 genes and two mutational genes as potential predictive biomarkers for TAVO412 efficacy. A prediction algorithm was then formulated based on the gene expression level. This predictor exhibited high accuracy in predicting efficacy in the CDX models and was further validated using the PDX model data.

## Materials and methods

### Test antibodies and reagents

The TAVO412 trispecific antibody was made in a stable CHO cell line, purified with Protein A and ion exchange chromatography, and tested using size exclusion chromatography (SEC) and capillary electrophoresis-sodium dodecyl sulfate (CE-SDS) (1). The amivantamab analogue was created in-house through controlled Fab-arm exchange (18), with its bispecific content confirmed using Bio Mix chromatography. The human IgG1 isotype null control antibody (isotype) was purchased from HAOKESAIYE (Beijing). In our study, the amivantamab analogue was produced in-house using the same amino acid sequences and glycosylation levels as amivantamab (sequences referred to World Health Organization Proposed INN List 121). Since this molecule was used for non-clinical research and was not the clinically-used amivantamab, we referred to this benchmark molecule as an amivantamab analogue for scientific accuracy.

### *In vivo* studies

Twenty-three cell lines were employed to establish CDX models. Eleven cell lines were procured from ATCC, namely NCI-H596, FADU, NCI-H358, BXPC3, SKHEP1, DMS79, NCI-H226, NCI-H1299, NCI-H460, HCC1806, and NCI-H1048. Nine cell lines were obtained from NANJING COBIOER, including KYSE150, SCC4, MDA-MB-231, BT20, A2780, TOV21G, HCC70, ASPC1, and EBC1. HCC827, NCI-H1975, and NCI-H292 were sourced from the National Collection of Authenticated Cell Cultures. All the cell lines were authenticated using short tandem repeat profiling and were tested for Mycoplasma contamination using the Myco-Lumi™ Luminescent Mycoplasma Detection Kit (Beyotime, #C0297M). Tumor cells were cultured in the corresponding medium with a 10% (v/v) fetal bovine serum (Gibco, #10091148) at 37°C in a 5% CO_2_. FADU and SKHEP1 cells were cultured in EMEM medium (ATCC, #30-2003); MDA-MB-231 and BT20 were cultured in DMEM medium (Gibco, #10566024). SCC4 and NCIH1048 were cultured in DMEM/F12 medium (Gibco, #10565042). TOV21G were cultured in completed medium purchased from COBIOER (#CBP20292M). EBC1 was cultured in MEM medium (Gibco, #41090036). All the other cells were cultured in RPMI-1640 medium (Gibco, #61870036).

Nine PDX were utilized, with four (LU2503, LU1901, LU5381, LU3075) performed by Crown Bioscience (Taicang, China), four (LU-01-1377, LU-01-1623, LU-01-0506, LU-01-1649) by WuXi AppTec (Suzhou, China), and one (LD1-0025-200717) by Lide Biotech (Shanghai, China).

The CDX and PDX models were developed in BALB/c nude mice (GemPharmatech Co., Ltd., China) or CB17 SCID mice (Beijing Vital River Laboratory Animal Technology Co., Ltd., China) by injecting 5-10 million tumor cells subcutaneously in the right flank for CDX models and tumor fragment (2-3 millimeter in diameter) for PDX models. Upon reaching a mean tumor volume in a range of 100–300 mm³, the tumor-bearing mice were randomized into various treatment groups, with the initiation of dosing designated on Day 0. TAVO412, amivantamab analogue (positive control), or an isotype antibody (negative control) were administered via intraperitoneal injection (IP) at a dose of 10 mg/kg, twice a week for a duration of 2 to 6 weeks. Tumor dimensions were regularly assessed using calipers to measure the length (L) and width (W). Tumor volume was calculated by 1/2 × length × width². Tumor growth inhibition (TGI%) was determined using the formula 100 × [1- (average tumor volume of the treatment group)/(average tumor volume of the control group)]. All animal experiments conducted in this study were approved by the Institutional Animal Care and Use Committee of the respective research institutions and facilities.

Statistical analysis was performed between the control and treatment group according to the tumor volumes at the last day of each study by two-tailed Student’s *t*-test. All statistical analyses as well as plotting were performed using GraphPad Prism 9.3.1. (GraphPad Software). A p<0.05 (between the control and the treatment group) was considered statistically significant. Asterisks indicated that the experimental p value was statistically significantly different from the associated controls at *p<0.05; **p<0.01; ***p<0.001; ****p<0.0001.

### Calculation of drug efficacy using exponential growth rate ratio

eGR quantified an overall tumor growth rate during the entire study duration based on the area under the time-growth curve (19, 20). eGR was the numerically equivalent to the rate constant of an exponential curve with equal area under the curve. Briefly, the area under tumor’s logarithm-transformed growth curve was calculated by the sum of the trapezoids and then subtracted by the rectangular area (time period x log TV_0_) of the baseline. Then the resulted area was normalized by ½ of squared observation period of time:


$$eGR_{i}=\;\frac{\sum_{j=1}^{J_{i}}\left(logTV_{j}+logTV_{j-1}-2logTV_{0}\;\right)\;*\;(T_{j}-T_{j-1})/2}{\frac{1}{2}\;{\left(T_{J_{i}}\right)}^{2}}$$

Where *i* was individual mouse i; *j* was individual measurement and *Ji* was the total number of tumor measurements for mouse i (except the measurement on day 0). *TV_j_* and *T_j_* were the tumor volume and specific study day in the j^th^ measurement for mouse i; *T_Ji_* was the total duration of the study for mouse i. For drug efficacy, we calculated the median of all possible ratios between the eGRs in the treatment group and the eGRs in the control group. We used the “get_Model_eGR” function in TuGroMix R package (version 1.1.0) to conduct this calculation (21).

### Whole-transcriptome sequencing

Formalin-fixed paraffin-embedded tumor samples from isotype group of 9 PDX models were utilized for RNAseq. Total RNA extraction followed the Qiagen Cat#73504 protocol. Concentration and quality of extracted RNA were measured using Qubit and 2100 Bioanalyzer, respectively. The library construction for RNAseq was performed with a matched kit (MGI, Cat:1000006384). Briefly, mRNA was captured and purified by MGIEasy rRNA Depletion Kit (MGI, Cat:100005953), followed by fragmentation using a fragment buffer. cDNA was synthesized following the processes: first strand cDNA synthesis, second strand cDNA synthesis; and cleanup. End-repair and cleanup were then performed to purify the double-stranded cDNA, followed by A-tailing, ligation of adapters, and cleanup. The DNA fragments with adapters were selected and amplified by a PCR. After cleaning up the product, a final library was qualified and quantified by Qubit and 2100 Bioanalyzer again before sequencing. A library with good concentration and fragment size was sequenced using the MGISEQ-2000RS. The final library was sequenced resulting in sequencing reading lengths of PE150.

The quality of RNAseq raw data was checked by FastQC software (https://www.bioinformatics.babraham.ac.uk/projects/fastqc/). The adapter and sequences with low quality were trimmed by Trimmomatic software (22). The reads were mapped to human(hg19) and mouse genome (mm10) by the STAR software (23). The data sets sorted after trimming and removing of mouse sequences were used for subsequent analyses. The reads were mapped to reference genes (ENSEMBL GRCh37.66) by the Bowtie software, and the gene expression was calculated by MMSEQ software (3). The gene expression values are represented as log2-transformed counts per million reads mapped (log2 CPM) or log2-transformed transcripts per million (log2 TPM).

The RNA-seq data of all CDX models was obtained from Cancer Cell Line Encyclopedia (CCLE) (24).

### Correlation analyses

The genes were first filtered based on the log2 TPM expression level, resulting in ~10,000 genes for downstream analysis. Spearman correlation coefficient and *p*-values were calculated for the correlation between each gene’s expression (in the unit of log2 TPM) and drug efficacy (eGR ratio) in the 23 CDX models in R (version 4.3.2). Genes were then sorted by *p*-values. A set of genes that had most strong positive or negative correlation with efficacy were used as the input to Enrichr (https://maayanlab.cloud/Enrichr/) to identify enriched pathways. The genes with biological relevance to cancer pathways were selected.

### Differential gene expression analysis

Differential gene expression analysis was carried out between the 9 responder and the 5 non-responder CDX models. The genes were first filtered with the criteria of CPM expression > 0.4 for each sample, and the resulting ~10,000 genes were normalized by the TMM method in the *edgeR* package (version 4.2.0) (25) in R (version 4.3.2). Gene counts were then transformed to log2 CPM, and the differential expression was fitted by linear regression. The resulting statistics were further moderated by the Empirical Bayes method (26) which estimated standard errors toward a global value, using the *limma* package (version 3.60.3) (27) in R. Genes showing the most significant p-values or large absolute log2 fold changes were submitted to Enrichr to identify genes related to target pathways. Moreover, genes closely associated with the target pathway, exhibiting an absolute log2 fold change greater than 1, were also selected.

### Driver mutation analysis

The driver mutations were selected using the whole exome sequencing (WES) data processing (28) and the WES data of the CDX models was obtained from CCLE. The driver mutation status was encoded as 0 when there was no driver mutation or 1 when there was one. Spearman correlation analysis was conducted for all the candidate genes that have driver mutation in at least one CDX model. The resulting p-values were ordered, and the genes were selected based on the p-values and their biological relevance to cancer.

### Linear regression analysis

Linear regression analysis was carried out using the “lm” function in R (version 4.3.2). The log2 TPM expressions of EGFR, c-Met, and VEGF-A, as well as the binary driver mutation status of EGFR in CDX models were used as the prediction variables, and the drug efficacy eGR ratio was used as the dependent variable in the multiple linear regression.

### Drug responsiveness prediction based on linear prediction score (LPS)

The development of drug responsiveness prediction model took a similar approach as previously published (29). Briefly, a t-statistic was calculated from the t-test of each predictor gene between 9 responder CDXs and 5 non-responder CDXs. The t-statistics were used as weights in the LPS summation. The LPS value was calculated for all the 23 CDX models with the equation shown in below, in which the gene expression value of log2 TPM (or driver mutation status 1/0) are preprocessed by Z-score normalization:


$$Z=\;\frac{X-\bar{X}}{\hat{s}}$$

Where *X* was the observed value; $\bar{X}$ was the estimated mean, and $\hat{s}\;$ was the estimated standard deviation.


$$LPS\left(X\right)=\sum_{j=1}^{K}a_{j}Z_{j}$$

The LPS was a linear combination of gene expression values, where $Z_{j}$ was the normalized gene expression of gene *j*, $a_{j}$ was a scaling factor represented by the t-value from t-test. K was the number of genes included in the prediction model. To predict the responsiveness to the drug in the PDX models, gene expression and driver mutation status in the PDX models were first preprocessed by Z-score normalization using the model developed from the training CDX data set. Then the LPS value of the PDX models was calculated, and the likelihood of being a responder or non-responder was calculated by applying Bayes’ rule:


$$\begin{array}{c} Probability\left(X\;in\;group\;1\right)\\ =\;\frac{\phi \left(LPS\left(X\right);{\hat{\mu }}_{1},{\hat{\sigma }}_{1}^{2}\right)\;}{\phi \left(LPS\left(X\right);{\hat{\mu }}_{1},{\hat{\sigma }}_{1}^{2}\right)+\phi \left(LPS\left(X\right);{\hat{\mu }}_{2},{\hat{\sigma }}_{2}^{2}\right)} \end{array}$$

where $\phi \left(x;\mu ,\;\sigma^{2}\right)$ represented the normal density function with mean μ , and variance $\sigma^{2}$ , and $\;{\hat{\mu }}_{1},\;{\hat{\sigma }}_{1}^{2},{\hat{\mu }}_{2},\;{\hat{\sigma }}_{2}^{2}$ were the observed mean and variance of the LPS values within response models (R) and non-response models (NR), respectively.

Log odds were alternate ways of expressing probabilities, therefore the probability was transformed to log-odds for better presentation:


Log−odds = ln (probability/(1−probability))

## Results

### TAVO412 exhibited variable levels of tumor growth inhibition across a panel of CDX models

**Figure 1:**
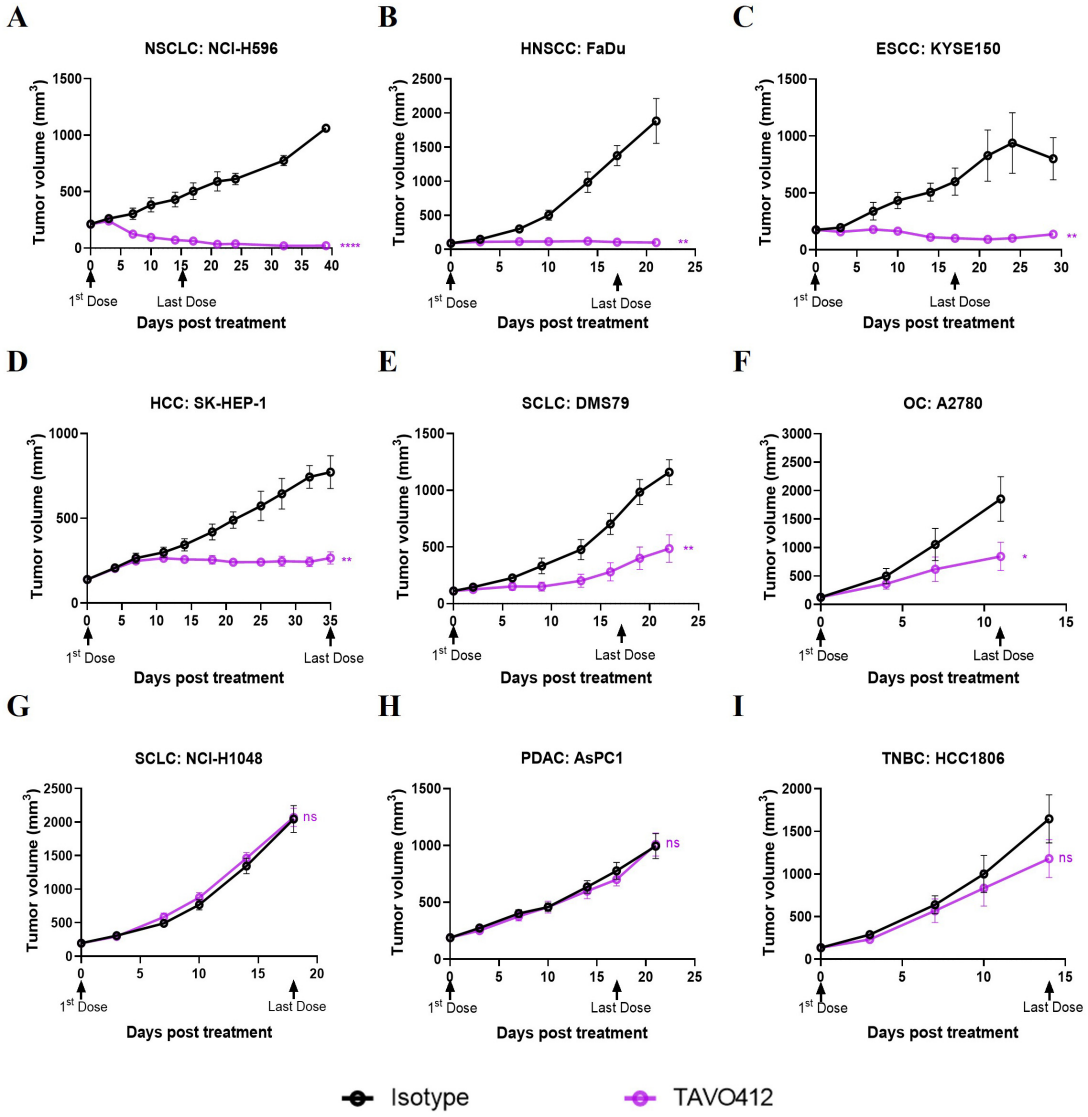
TAVO412 demonstrated anti-tumor activities in tumor cell-line-derived xenograft models (representative data). Immunodeficient mice were subcutaneously inoculated with corresponding tumor cells. Tumor bearing mice were randomized and treated with 10 mg/kg TAVO412, or isotype control (i.p., BIW; black arrows indicated the first and last dosing days). Tumor growth was monitored twice weekly. The black points and curves profiled the Isotype mAb; the violet points and curves profiled the TAVO412. The models were categorized into 3 levels based on the responses to TAVO412: **(A-C)** Response models (R, TGI ≥ 70%), **(D-F)** Partial Response models (PR, 30% ≤ TGI < 70%) and **(G-I)** Non-Response models (NR, TGI < 30%). Statistical comparison (student’s t-test) run for each treatment group as compared to isotype control (ns, not significant; *p<0.05; **p<0.01; ****p<0.0001). For all graphs, the error bars represented standard error of the mean (SEM). The abbreviations were: NSCLC, non-small cell lung cancer; HNSCC, head and neck squamous cell carcinoma; ESCC, esophageal squamous cell carcinoma; HCC, hepatocellular carcinoma; SCLC, small-cell lung cancer; OC, ovarian cancer; PDAC, pancreatic ductal adenocarcinoma; TNBC, triple negative breast cancer; BIW, twice-weekly.

The antitumor activities of TAVO412 were tested in a total of 23 CDX models, including 9 NSCLC models, 4 TNBC models, 3 HNSCC xenografts, 2 xenografts for each of SCLC, PDAC, and OC, and 1 xenograft of HCC (**Supplementary Table 1**). The tumor-bearing mice were treated with TAVO412 or isotype control at 10 mg/kg when the tumors were well established. For developing the biomarker profiles, the responses in these CDX models to TAVO412 were classified into 3 categories - Responder (R, TGI ≥ 70%), Partial Responder (PR, 30% ≤ TGI < 70%), and Non-Responder (NR, TGI < 30%). Of the 5 out of 9 NSCLC models, all 3 HNSCC models, and 1 of 2 PDAC models showed TGI values greater than 70% and were categorized as responder cell lines to TAVO412 (representative growth curves shown in **Figures 1A–C** and TGI in **Supplementary Table 1**). Three out of 9 NSCLC models, 2 out of 2 OC, 2 out of 4 TNBC, 1 out of 2 SCLC and I HCC model exhibited partial responses to TAVO412 with the TGI values in the range of 36% to 66% (representative growth curves shown in **Figures 1D–F** and TGI in **Supplementary Table 1**). However, there were five models were binned as non-responders to TAVO412 since they had TGI values less than 30%, including 2 out of 4 TNBC cell lines, 1 out of 9 NSCLC, 1 out of 2 SCLC, and 1 out of 2 pancreatic cancer cell lines (representative growth curves shown in **Figures 1G–I** and TGI in **Supplementary Table 1**).

In summary, we have shown for the first time the strong to moderate antitumor activities of TAVO412 in HNSCC, OC, HCC and SCLC xenografts, in addition to the reported effective data on NSCLC, GC, PDAC and TNBC models. These *in vivo* profiles supported the potential utility of TAVO412 to treat patients with these types of cancer. However, not all models within a particular indication responded equally well to TAVO412. The diverse responses to TAVO412 highlighted the necessity of developing a biomarker strategy to assist in identifying patients who could be more likely to derive benefit from TAVO412 treatment.

### TAVO412 effectively suppressed tumor growth in NSCLC PDX models with various mutations

NSCLC patients with EGFR and c-Met mutations were the primary patient population that could benefit from TAVO412 treatment. PDX models of lung cancers bearing a variety of mutations were considered to be more clinically relevant preclinical models than CDX models. Therefore, we assessed the tumor inhibition effect of TAVO412 in PDX models of NSCLC that represented diverse genetic mutation scenarios that could be found in patients. These mutations included EGFR activating mutations in the cytosolic domains encoded in exons 18 to 21. Point mutations in exon 18 (G719A and E709A), in exon 20 (T790M and C797S), and in exon 21 (L858R) were present in at least one of the PDX models. In addition, exon 19 deletion and exon 20 insertions were also identified in the samples. The activation of the MET pathway resulting from overexpression, gene amplification, and MET exon 14 skipping mutations was also represented by the PDX models. The antitumor activities of TAVO412 were evaluated in this panel of PDX models harboring sensitive or resistant EGFR mutations as well as models having c-Met amplification or exon 14 skipping mutations (**Supplementary Table 2**). A SCLC model (LU-01-1377) having EGFR L858R mutation and MET over-expression was also tested along with the 8 NSCLC models.

**Figure 2:**
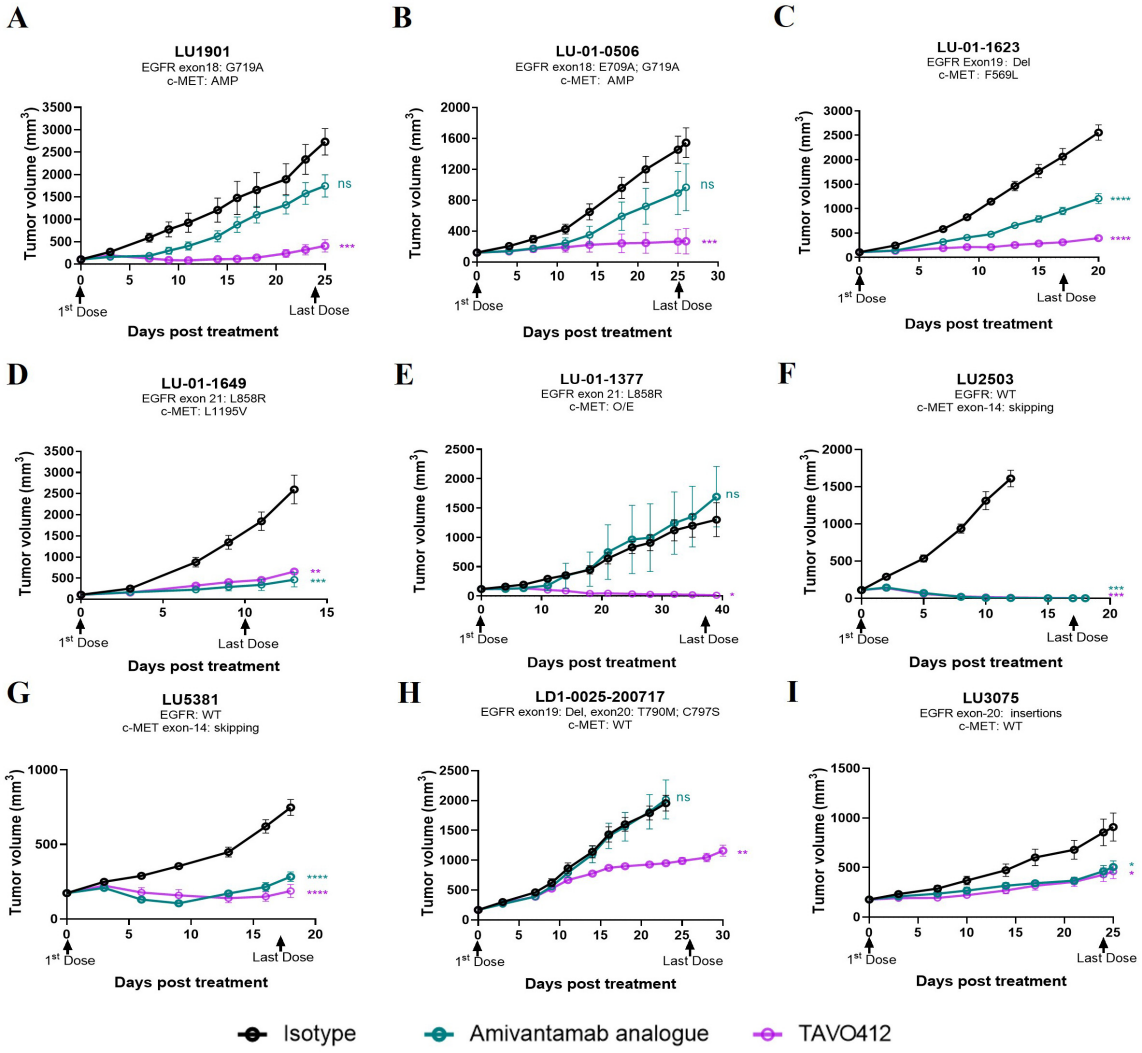
TAVO412 demonstrated anti-tumor activities in patient-derived xenografts carrying EGFR and c-Met mutations. **(A–I)** Female BALB/c nude mice were subcutaneously inoculated with corresponding tumor fragments. Tumor bearing mice were randomized and treated with 10 mg/kg TAVO412, amivantamab analogue, or isotype control (IP – BIW; black arrows indicated the first and last dosing days). The black points and curves profiled the Isotype mAb; the violet points and curves profiled the TAVO412; and the blue points and curves profiled the amivantamab analogue. Tumor growth was monitored twice weekly. Statistical comparison (student’s t-test) run for each treatment group as compared to isotype control (ns, not significant; *p<0.05; **p<0.01; ***p<0.001; ****p<0.0001). For all graphs, error bars represent SEM. The abbreviations were: AMP, amplification; O/E, overexpression; WT, wild type; BIW, twice-weekly.

TAVO412 demonstrated strong antitumor activities in five PDX models with drug-sensitive EGFR mutations, including exon 18 G719A and E709A (LU1901 and LU-01-0506), exon 19 Del (LU-01-1623), and exon 21 L858R mutations (LU-01-1649 and LU-01-1377), achieving TGI in the range of 75% to 99% (**Figures 2A–E**, **Supplementary Table 2**). The tumor growth control effects of TAVO412 in these five models were superior when compared to the amivantamab analogue at the same dose level, except in the LU-01-1649 model, which showed comparable tumor growth inhibition effects between the two antibodies (**Figure 2D**). LU-01-1377, a SCLC having an EGFR L858R mutation, exhibited high sensitivity to TAVO412 treatment compared to amivantamab analogue, whereas the amivantamab analogue was not efficacious (**Figure 2E**). TAVO412 exhibited comparable antitumor activity with the amivantamab analogue in two models bearing MET 14 skipping mutations (LU2503 and LU5381), achieving TGIs of 99% and 75%, respectively (**Figures 2F, G**, **Supplementary Table 2**). The PDX model LDI-0015200717, harboring both T790M and C797S EGFR mutations conferring resistance to EGFR-TKIs, still responded to TAVO412 treatment, with a TGI of 51%, while the amivantamab analogue showed no antitumor activity (**Figure 2H**, **Supplementary Table 2**). The LU3075 model, carrying an EGFR exon 20 insertion mutation (P772_H773insDNP), exhibited poor responses to EGFR-TKIs and cetuximab (30). TAVO412 and the amivantamab analogue inhibited tumor growth of LU3075 model achieving a TGI of 49% and 44%, respectively (**Figure 2I**, **Supplementary Table 2**).

In conclusion, all nine PDX models exhibited positive responses to TAVO412 treatment, demonstrating comparable or stronger antitumor efficacy compared to the amivantamab analogue. These results portend the therapeutic promise of TAVO412 in addressing a spectrum of patients with varying genetic abnormalities of EGFR and c-Met, regardless of their sensitivity or resistance to currently utilized EGFR-TKIs or amivantamab.

### Identification of predictive genes for TAVO412 response in the CDX models

**Figure 3:**
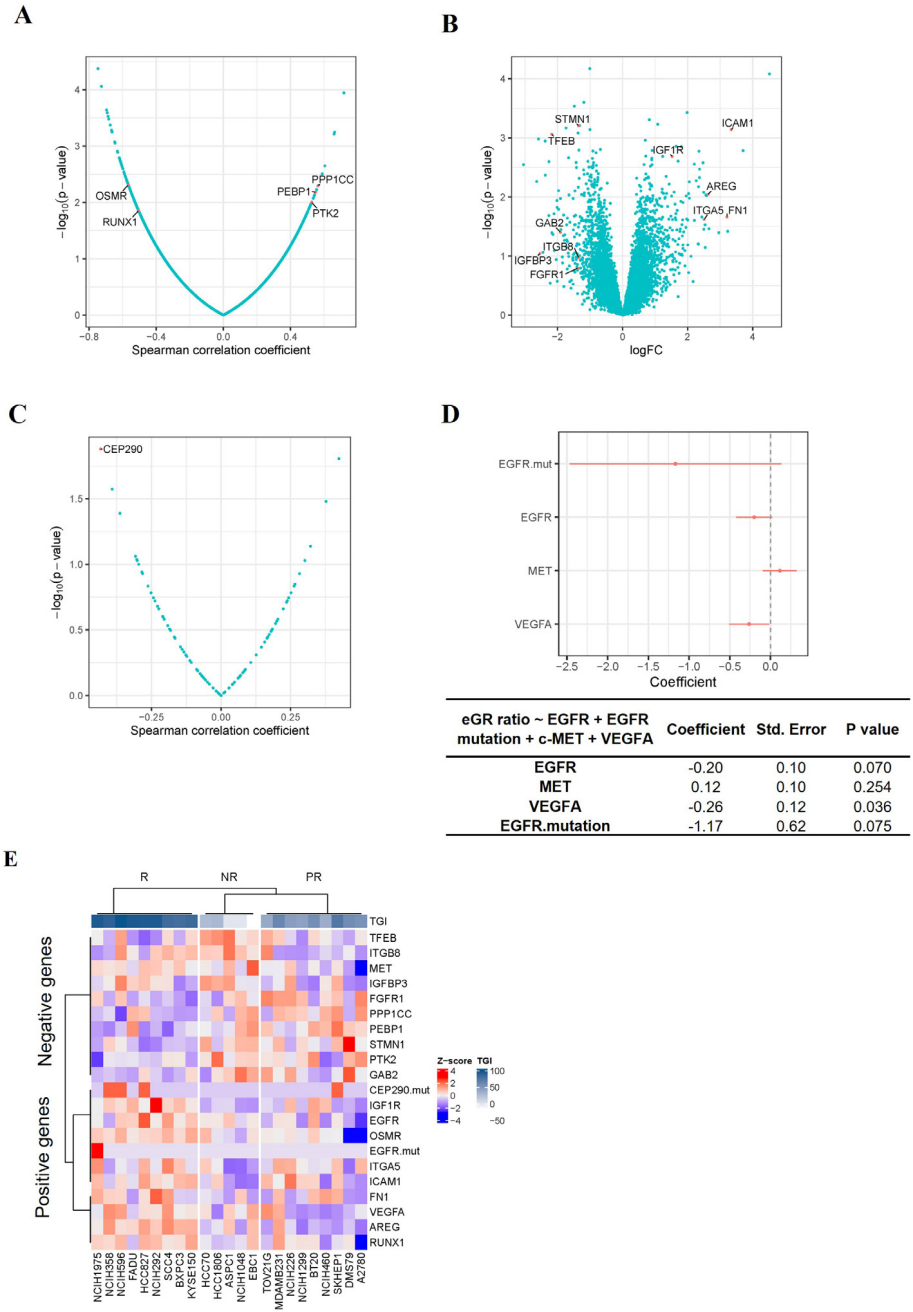
Identification of TAVO412 Predictive Genes in CDX Models. **(A)** Volcano plots of Spearman correlation (x-axis) and significance (y-axis) evaluated between drug sensitivity (eGR ratio) and the expression of specific genes; Genes conferring enhanced TAVO412 sensitivity were depicted on the left: *oncostatin M receptor (OSMR) and Runt-related transcription factor 1 (RUNX1);* Genes conferring resistance are depicted on the right: *serine/threonine-protein phosphatase PP1-gamma catalytic subunit* (*PPP1CC*), *Phosphatidylethanolamine-binding protein 1(PEBP1) and nonreceptor protein tyrosine kinase 2 (PTK2);* **(B)** Volcano plots based on log2 fold-change of gene expression (x axis) against -log10 p-value (y axis) showing the proportion of differentially expressed genes between the responders (9 CDXs) and non-responders (5 CDXs); Genes conferring enhanced TAVO412 sensitivity were depicted on the right: *Intracellular adhesion molecule-1 (ICAM-1), Insulin-Like Growth Factor 1 Receptor (IGF1R), Amphiregulin (AREG), Fibronectin 1 (FN1) and Integrin Subunit Alpha 5(ITGA5); Genes conferring resistance are depicted on the left: Stathmin 1(STMN1), Transcription factor EB (TFEB), GRB2 Associated Binding Protein 2(GAB2), Integrin Subunit Beta 8 (ITGB8), Insulin-Like Growth Factor Binding Protein 3(IGFBP3) and Fibroblast Growth Factor Receptor 1(FGFR1);* **(C)** Volcano plots of Spearman correlation (x-axis) and significance (y-axis) evaluated between drug sensitivity (eGR ratio) and the driver mutation. The mutation of the gene *Centrosomal Protein 290 (CEP290)* conferred TAVO412 sensitivity; **(D)** The coefficients of each drug target gene expression and EGFR mutation on the drug efficacy (eGR ratio) from multiple linear regression. The red bars denoted a 95% confidence interval. The coefficient, standard error and p values for the coefficients for each independent variable was listed in the table below. The expression of *Vascular Endothelial Growth Factor A (VEGF-A) and Epidermal Growth Factor Receptor (EGFR), plus EGFR* mutation predict TAVO412 effectiveness, while *MET Proto-Oncogene (c-Met)* gene conferred resistance; **(E)** Heatmap illustrating the expression levels of the identified predictive genes across 23 CDX models, categorized according to their response to TAVO412 (R, response models; PR, partial response models; NR, non-response models). The color gradient for normalized expression values (Z-scores) and tumor growth inhibition (TGI) values were shown. For TGI range, the colors ranged from white (less responsive) to dark blue (more responsive); for the expression levels, the colors ranged from red (high Z-scores) to blue (low Z-scores).

Since TAVO412 had a range of TGI in the panel of CDX models, we investigated which gene expression profiles could be associated with the responses to TAVO412. Identification of correlations could assist in identifying patients that could be sensitive to TAVO412 treatment in the clinic. To implement this, the individual gene expression levels of ~10,000 genes were analyzed for correlation with efficacy endpoints. A group of genes that had the strongest positive or negative correlations with efficacy were used as the input to the Enrichr that could determine gene enrichment in pathway databases. The eGR ratio of treated group to the PBS control was used to serve as the efficacy endpoint instead of TGI to better capture the dynamics of tumor growth in the animal models as compared to TGI that only accounted for the data on the last day (20). The efficacy results expressed in percent TGI based on the tumor volume on the last data of the study were plotted against eGR ratio and the two efficacy indices demonstrated very good inverse correlation with a correlation coefficient of -0.94 (p<0.0001) (**Supplementary Figure 1**). A total of five genes were identified with a Spearman correlation coefficient of < -0.4 or > 0.4 (p<0.05), with 2 genes (*OSMR* and *RUNX1*) predicting the efficacy of TAVO412, and 3 genes (*PTK2*, *PEBP1* and *PPP1CC*) predicting the resistance to TAVO412 (**Figure 3A**). Secondly, differentially expressed genes between the responder (9 CDXs) and non-responder models (5 CDXs) were investigated. Eleven genes having at least a two-fold difference in gene expression between the R and NR models were identified; with 5 genes (*FN1, ICAM1, AREG, ITGA5 and IGF1R*) predicting the response to TAVO412; and 6 genes (*STMN1, GAB2, IGFBP3, ITGB8, FGFR1 and TFEB*) conferring resistance (**Figure 3B**). Thirdly, we performed a correlation analysis between all the annotated cancer driver mutations (28) and the eGR ratios and found that the CEP290 mutation had a significant correlation for TAVO412 efficacy (**Figure 3C**). Lastly, since EGFR, c-Met, and VEGF-A were the 3 targets of TAVO412, we assessed their correlation to drug efficacy by a multiple linear regression model, in which the gene expression levels of the three genes as well as EGFR mutation status were served as the independent variables, and eGR ratio as the dependent variable. This linear model explained 39% of the variance in eGR ratio (R^2^ = 0.39), while VEGF-A expression was significantly negatively correlated with eGR ratio (**Figure 3D**). For every increase of VEGF-A expression (in units of log_2_TPM) while holding the other three independent variables constant, eGR was expected to decrease by 0.26. EGFR expression and EGFR mutation status were on the border of significance for the associations. Interestingly, the c-Met expression had a positive correlation with the eGR ratio (negatively correlated with efficacy), although the *p* value was not significant. The target gene expression level plus EGFR mutation were included in the final prediction gene set, although none was top ranked in either correlation analysis or differentially expressed gene analysis.

In total, a predictor comprising of the expression level of 19 genes plus mutational status of 2 genes was obtained to predict the efficacy of TAVO412, with 9 genes and 2 mutational genes for TAVO412 sensitivity and 10 genes conferring resistance. The relationship of the gene expression levels and the TGI of the 23 CDXs was illustrated in a heatmap by clusters (**Figure 3E**). The TAVO412 sensitive models (R group) predominantly showed high expression of genes predicting positive outcomes, while the resistant models (NR group) showed higher expression of negatively predicting genes and vice versa (**Figure 3E**, **Supplementary Figure 2A**). The gene expression status of the partial response models (PR group) fell in between that of the sensitive and resistant models (**Figure 3E**, **Supplementary Figure 2A**).

### Formulation of a prediction model

Since the transcriptomic make-up was more complicated than simplified responder or non-responder models, we developed a method to quantitatively describe the gene expression levels of the identified gene panel and to predict the probabilities of being responsive to the therapy. We therefore harnessed a previously reported prediction model – LPS, which is a linear combination of gene expression values (29), to compute a score for each sample by summing up the selected gene expression profiles. The weight of each gene in the LPS model was the t-statistic from t-test assessments (See Methods and **Supplementary Figure 2B**). The probability of being sensitive to TAVO412 treatment was estimated by the relative proximity of the sample’s LPS score to the LPS distribution of the R versus NR groups of the 23 training CDXs (see details in Materials and Methods).

**Figure 4:**
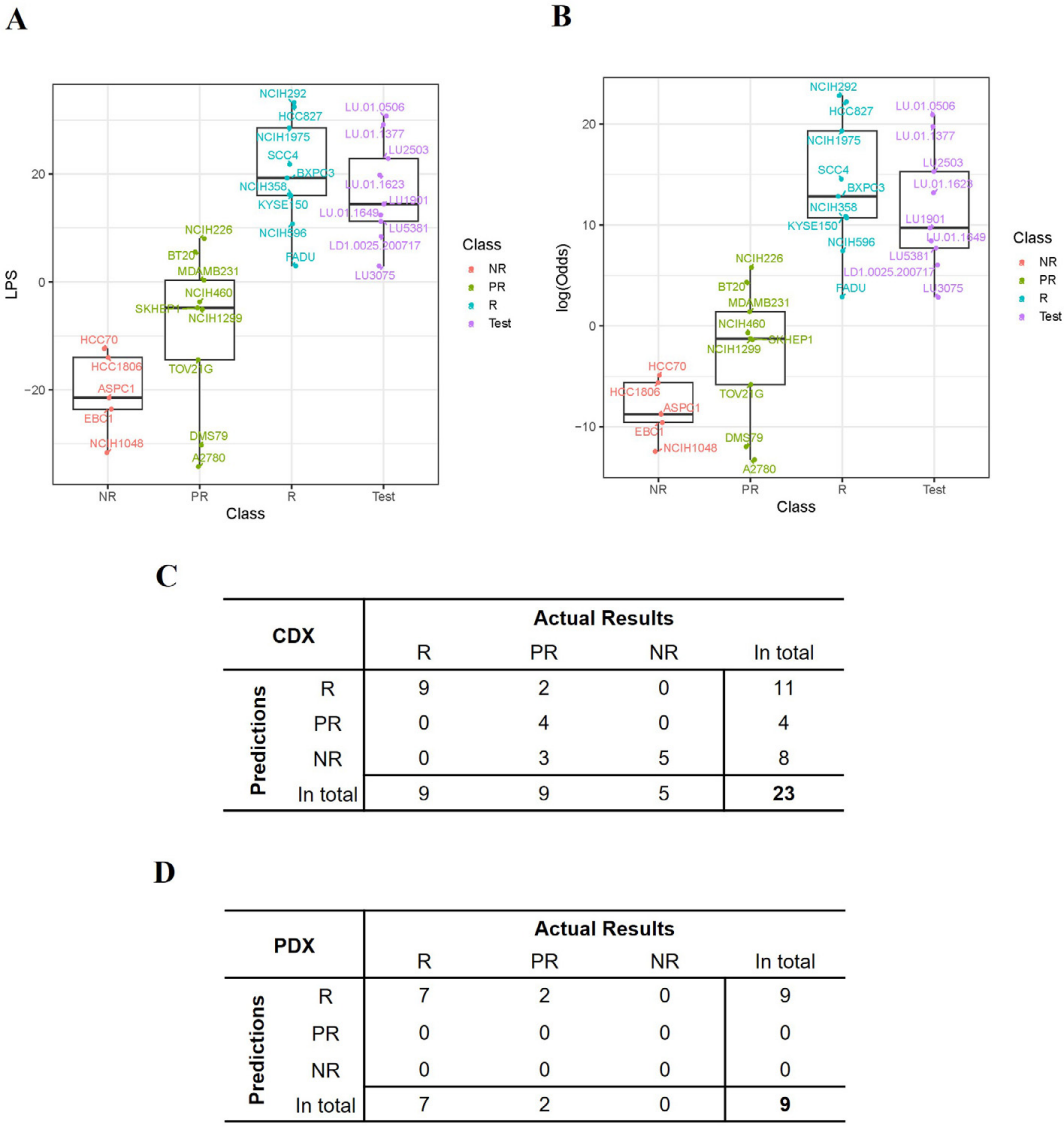
Performance of the TAVO412 efficacy predictor. **(A)** The expression score (LPS) was computed for each model based on the expression levels of the selected 21 genes and the distribution of the LPS scores for all the 23 training CDX values and 9 PDXs was listed. **(B)** The probability that a model was responsive to TAVO412 treatment was calculated for all the 23 training CDXs and 9 PDXs and was transformed to log-odds shown on y-axis. A 90% probability (corresponding to log-odds = 2.2) was used as a cutoff to define the testing PDXs’ responsiveness to TAVO412 treatment; The CDX and PDX classifications were labeled in the x-axis as NR, non-responsive (red); PR, partial-responsive (yellow); R: responsive (blue); Test: PDXs (violet); **(C)** The table contrasted the actual results of the assignments of the CDX training model versus the TAVO412 efficacy predictor. The assignments were classified into the R, PR, and NR subgroups. **(D)** The table contrasted the actual results of the assignments of the PDX testing model versus the TAVO412 efficacy predictor. The assignments were classified into the R, PR, and NR subgroups. The abbreviations were: LPS, linear prediction score; PDX, patient-derived xenograft; CDX, cell line-derived xenograft; R, responsive models; PR, partial-responsive models; NR, non-responsive models.

The LPS values and the probability (represented by log-odds as described in method) of being drug-sensitive of the 23 training CDXs were shown in **Figures 4A, B**. Three groups of models were largely clustered based on the responsiveness to TAVO412, with the responder models having the highest LPS score, the non-responder models showing the lowest LPS score (**Figure 4A**). The LPS values were significantly correlated with the eGR ratios with a Spearman’s Rho coefficient of -0.79 (**Supplementary Figure 3**). From the probability assessment (**Figure 4B**), all the 5 NR and 9 R models were correctly predicted, while 4 out of 9 PR models were correctly predicted, with three PR models (DMS79, A2780 and TOV21G) underestimated and 2 PR models (BT20 and NCI-H226) overestimated using a cutoff of 90% probability to decide final subgroup membership (response models: Probability ≥ 90%; partial response models: 10% ≤ Probability <90%; non-response models: Probability < 10%) (29). Therefore, the accuracy for the training model set was 78% (18 out of 23 models were correctly predicted; **Figure 4C**).

### Validation of the predictor training set from the PDX models

The predictive power within the training sets was anticipated, given that half of the genes (11 out of 21) were selected from the analysis of the differential expressed genes between R and NR models. The selection process resulted in the distinct separation of R versus NR sub-groups in the LPS score graph, while the prediction for several of the PR models did not deviate far from the actual responses. To further verify the strength of the predictor developed based on the training set, we validated the prediction algorithm using the results from the 9 PDX models. The responses to TAVO412 in the 9 PDX models are shown in **Figure 2**. The LPS scores of these PDX model were calculated and plotted to reveal that the 9 PDXs fell within the range of responder CDX values (**Figure 4A**). Subsequently, the probability of response to TAVO412 treatment was calculated, and the results showed that 9 out of 9 PDX models were predicted to be responsive to TAVO412. All were correct except for LD1.0025.200717 and LU3075, which were overestimated since they were partial response models (**Figure 4B**). Consequently, the prediction accuracy of the validation PDX set showed the similar accuracy compared to the training CDX model data set with an accuracy of 78% (7 out of 9 models were correctly predicted; **Figure 4D**).

## Discussion

TAVO412 inhibited tumor growth in most of the CDX and PDX models of NSCLC (**Figures 1**, **2**, **Supplementary Tables 1**, **2**). Such a result was expected since EGFR was the key driver in NSCLC progression. PDX models showed superiority in recapitulating the molecular, genetic, and histological heterogeneity of the original human tumor, thus holding greater translational value than the CDX models (31). Therefore, it was encouraging to observe that TAVO412 was particularly effective in the PDX models bearing EGFR exon 18 mutations, exon 19 deletion, exon 21 L858R mutation, and c-Met exon-14 skipping mutations (**Figure 2**). Notably, TAVO412 exhibited superior antitumor activity compared to amivantamab in the PDXs bearing exon 18 mutation (LU1901 and LU-01-0506) and exon 19 deletion (LU-01-1623), suggesting a potential tumor control effect in patients that did not fully respond to amivantamab (**Figures 2A–C**). Additionally, TAVO412 mediated a near complete regression of an SCLC tumor – LU-01-1377, which harbored an L858R mutation (**Figure 2E**). SCLC with EGFR gene mutation typically manifested as a transformation occurring after EGFR tyrosine kinase inhibitor therapy, because primary SCLC showing EGFR mutation was rare. Indeed, the patient from whom the LU-01-1377 tumor originated had a history of osimertinib treatment, and the LU-01-1377 xenograft tumor model exhibited resistance to EGFR-TKI inhibitors (erlotinib, afatinib, and osimertinib; data not shown). This profile led to a hypothesis that the patient could have undergone a transformation from NSCLC to SCLC, further highlighting the potential utility of TAVO412 in such a subgroup of SCLC patients. Both TAVO412 and amivantamab displayed moderate antitumor activities in the model with exon 20 insertion (LU3075) (**Figure 2I**). TAVO412 demonstrated moderate tumor inhibition in a PDX model with both T790M and C797S mutations (LDI-0015200717), whereas amivantamab had no effects on this tumor (**Figure 2H**). With HNSCC driven by EGFR aberrations, all three CDX models exhibited sustained tumor growth control by TAVO412 even after treatment cessation (**Figure 1B**, **Supplementary Table 1**). The profile of TAVO412 tumor growth inhibition effects in animal models of OC, HCC, and SCLC were described in **Figure 1** and **Supplementary Table 1**.

This rich *in vivo* antitumor dataset allowed sophisticated quantitative correlation analyses to develop transcriptomic biomarkers. In addition, a separate set of PDX models created an opportunity to cross validate the prediction algorithm. A systematic transcriptomic analysis identified a gene expression signature strongly predictive for solid tumors that were highly susceptible to TAVO412 treatment. The therapeutic responses from the 23 training CDX models to TAVO412 treatment were strategically linked to individual gene expression levels. We focused on four types of predictive gene profiles: individual genes that correlated with the responses either positively or negatively (**Figure 3A**); genes that differentially expressed between responders and non-responders (**Figure 3B**); gene mutations that served an indicator of the responses (**Figure 3C**); and genes that directly related to TAVO412 target engagement (expression of EGFR, c-Met, VEGF-A and EGFR mutation; **Figure 3D**). The resulting set of 19 gene expression profiles plus two gene mutations formed the final predicting gene sets (**Figure 3E**). Using the gene expression levels, an LPS score was calculated for each tumor sample set to predict its probability of response (**Figures 4A, B**). The prediction model achieved a 78% accuracy in the training model data set (**Figure 4C**). Furthermore, the model also predicted the PDX models well in the validation run, resulting in similar accuracy of 78% (**Figure 4D**). Since there were no non-responders in the PDX models, the validation for prediction of NRs may require more data.

In order to enhance the potential of predictability, the selection of the final 21 genes was not only based on the strength of their performance but also on their association with the target pathways of TAVO412 since a subset of the markers could always randomly correlate with some parameters being analyzed when handling multiplexed ‘big’ data (32). We selected those biomarkers first by screening genes by correlation analyses, then applied gene ontology and pathway analyses of all top ranked genes, and finally manually selected specific genes that were in the relevant pathways. Hence, we presented a combination of objective (statistical screening and gene ontology analyses) with known signaling biology in the literature. While, these genes would be considered not to be specific and off-target to TAVO412, we presented the results from multiple assessments to show that these “unspecific genes” could have some role. The rationale behind the positive or negative predictive nature of these genes was investigated (**Supplementary Tables 3**, **4**) thoroughly for the decision making. Circulating VEGF-A (gene: *VEGF-A*) concentration was a predictive biomarker for bevacizumab in breast, pancreatic, and gastric cancers (33). Similarly, EGFR (gene: *EGFR*) expression, mutation status, and amplification were predictive biomarkers for cetuximab in both clinical and preclinical settings (20, 34, 35). Insulin-like growth factor 1 receptor (IGF1R) has been identified as a mechanism of resistance to EGFR-TKIs. However, its frequent co-expression with EGFR across various cancer types may contribute to its designation as a positive predictor (36, 37). Similarly, Oncostatin M receptor (gene: *OSMR*), a member of the type I cytokine receptor family, functions as a co-receptor for EGFR and enhanced EGFR signaling in glioblastoma (38). Therefore, both IGF1R and OSMR expression could correlate with the expression of EGFR. Amphiregulin (gene: *AREG*), an EGFR ligand, could stimulate EGFR for a longer time when compared to EGF‐stimulated EGFR. AREG engagement could result in higher levels of activated EGFR protein from either enhanced receptor stabilization or increased recycling to the cell surface (39). Fibronectin 1 (gene: *FN1*), one of the key genes upregulated by AREG-stimulated EGFR pathway. Higher levels of FN1 mRNA and protein was observed in breast cancer cell lines that also expressed high levels of AREG (40). The *ITGA5* gene encoded the integrin subunit α5 that could interact with ITGB1 to generate integrin α5β1 that was a receptor for FN1 (40). Therefore, while AREG was a ligand of EGFR, and both fibronectin 1 and integrin α5β1 are the downstream effector molecules of AREG stimulated EGFR; thus, their high expression levels could indicate the activation of EGFR signal pathway. Additionally, intracellular adhesion molecule-1 (gene: *ICAM-1*) was a downstream molecule that could be up-regulated by the PI3K/Akt signaling pathway through AREG activation of EGFR (41). The *RUNX1* gene encoded runt-related transcription factor 1 (RUNX1), which could promote the increased EGFR phosphorylation and could upregulate EGFR transcription level by directly binding to the *EGFR* gene promoter (42). Therefore, our profiling showed that all those positive type genes had a positive association with EGFR pathways in tumor cells, either through co-expression or being upstream or downstream effector molecules of EGFR pathways. The role of CEP290 gene in cancer was described to have crucial roles in the proliferation, migration, infiltration, and ferroptosis of hepatocellular carcinoma (HCC) tissues and various liver cancer cell lines (43). *IGF1R* and *ICAM1* were the top two among those positively predicting genes, carrying the most weight in the calculation of the LPS score (**Supplementary Figure 2B**).

There were ten genes negatively predicting responses to TAVO412 treatment (**Supplementary Table 4**). Five genes have been reported to play a role in conferring EGFR-TKI resistance. Fibroblast growth factor receptor 1 (gene: *FGFR1*) mediates a well-known alternative pathway that complemented both EGFR and VEGF signaling, thus conferring resistance to therapies targeting EGFR or VEGF (35, 44). Nonreceptor protein tyrosine kinase 2 (gene: *PTK2*), stathmin1 (gene: *STMN1*), GRB2-associated-binding protein 2 (gene: *GAB2*), and integrin beta-8 (gene: *ITGB8*) have been reported to induce EGFR-TKI resistance in preclinical experiments (45–48). Additionally, phosphatidylethanolamine-binding protein 1 (gene: *PEBP1*) *(* 49) and serine/threonine-protein phosphatase PP1-gamma catalytic subunit (gene: *PPP1CC) (* 50) were selected for negative prediction, albeit there was no evidence showing their role mediating the resistance to EGFR targeting therapies so far. Interestingly, c-Met, one of the targets of TAVO412, which was supposed to be a positive predictor, showed a trend of negatively predicting the response, although the association between gene expression and eGR ratio was not significant (**Figure 3D**). Consistent with our findings, the clinical significance of c-Met overexpression as a useful biomarker for MET-targeted therapies was not yet clearly established (51). Another gene showing confounding results was transcription factor EB (gene: *TFEB*), which was a master regulator of lysosomal function and autophagy. TFEB could increase the cytotoxicity of anti-EGFR in *in vitro* assays, by recovering the degradation process of EGFR under hypoxic conditions (52). Besides, there were conflicting reports about the role of the insulin-like growth factor-binding protein 3 (gene: *IGFBP3*) in EGFR-TKI resistance. On one hand, IGFBP3 could act as a suppressor of IGF1R signaling by binding to IGF1R ligands. Since IGF1R signaling was an alternative pathway to EGFR, the downregulation of IGFBP3 was associated with EGFR-TKI resistance (53). On the other hand, increased IGFBP3 expression could lead to afatinib resistance by enhancing IGF1R activity and subsequent AKT phosphorylation in PC-9 cell line (54). Those genes that had not elucidated a clear mechanistic relationship or had contradictory information were incorporated in the prediction algorithm based on their empirical values. *TFEB* and *STMN1* ranked as the two most significant negatively predicting genes, having the greatest influence on the LPS score calculation. (**Supplementary Figure 2B**).

In summary, we identified genes linked to tumor associated target pathways that had utility in predicting the efficacy of TAVO412. Despite some genes lacking a direct link to EGFR, c-Met, or VEGF engagement, their biologic role in cancer development and response to TAVO412 treatment remains to be elucidated. Furthermore, the prediction gene sets and the computational algorithm, developed based on the animal data, could be further adjusted with more data available from both preclinical and clinical settings. Besides, developing a companion diagnostic (CDx) could facilitate the application of the predictor for favorable patient identification in the clinic. Despite these 21-gene profiling that could serve as biomarkers, there were several limitations to this study. We presented the efficacy of TAVO412 using xenograft models in immunodeficient nude mice. The rationale for using these xenograft models to assess TAVO412 efficacy was to illustrate the three major mechanisms of action: inhibition of EGFR/c-MET signaling, Fc effector functions (ADCC, ADCP and CDC), and control of VEGF-A-induced angiogenesis. The direct tumor inhibition effect by EGFR/c-MET signaling blockade was presented in xenograft efficacy models of the benchmark molecule amivantamab (55). Immunodeficient mice that include nude and CB17 SCID mice retain NK cells and macrophages, which can mediate ADCC and ADCP activities, respectively, through human antibodies (56). Human IgGs exhibited binding strengths to mouse FcγR that are comparable to those observed with human ortholog receptors (57). Antibody Fc-CDC activity has been demonstrated in CB17 SCID mice (58). However, research on the CDC activity of human IgGs in mouse models is limited. There is no definitive conclusion regarding the effectiveness of evaluating CDC activity using mouse complement systems. Human tumor-derived VEGF can be systemically blocked using specific antibodies, such as TAVO412 and avastin, resulting in significantly suppressed tumor growth in mouse models (59). TAVO412 only suppressed tumor-derived human VEGF-A, but not the host-derived mouse VEGF-A, which was secreted from mouse fibroblast and immune cells that surrounded the tumor mass supporting residual angiogenesis and growth (59, 60). Therefore, the function of anti-VEGF domain of TAVO412 may not be fully evaluated in preclinical models.

Considering the complexity of cancer biology, the treatment responses could be influenced by other factors beyond gene expression. In addition, there could be resistance mechanisms linked to the crosstalk between targeted pathways. Since preclinical models may not fully replicate the human tumor microenvironment, the clinical translation of these findings could be limited. Caution is warranted in interpreting the prognosis prediction algorithm derived from these animal studies. Therefore, further validation of these biomarkers would be required for patient selection to guide evaluation of TAVO412 clinical efficacy.

## Data Availability

Datasets are available on request: The raw data supporting the conclusions of this article will be made available by the authors, without undue reservation. The RNAseq data presented in the study are deposited in the Sequence Read Archive (SRA) repository, accession number PRJNA1218197.
