# Supplementary material for: Developing transcriptomic biomarkers for TAVO412 utilizing next generation sequencing analyses of preclinical tumor models
Source: Front Immunol. 2025 Feb 10;16:1505868. doi: 10.3389/fimmu.2025.1505868 (PMC11847686; doi:10.3389/fimmu.2025.1505868)
Supplement: Supplementary file 1 [file DataSheet1.docx]

Supplemental Figure 1


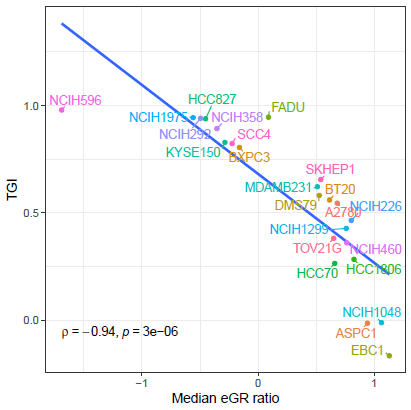


**Supplementary Figure 1. Correlation of eGR ratio with TGI**

The correlation between the two anti-tumor efficacy endpoints – the eGR ratio and TGI – across 23 CDX models was tested. The calculations for the two endpoints were detailed in the methods section. The x-axis represented the values of the median eGR ratio and the y axis represented the TGI values for each model. Each model was represented by a specific color for better separation. A significant inverse correlation was evident, with a Spearman correlation coefficient of -0.94 (*p* < 0.0001), indicating a strong negative association between the two endpoints. The abbreviations were: eGR, exponential growth rate; TGI, tumor growth inhibition; CDX, cell line-derived xenograft.

Supplemental Figure 2

**A**


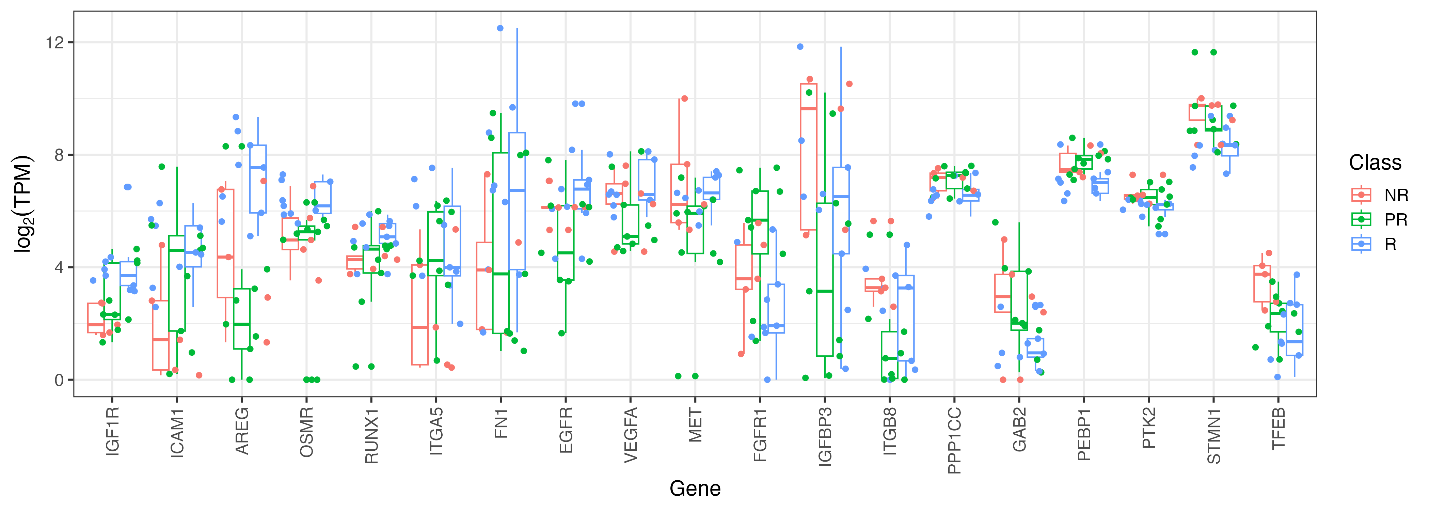


**B**


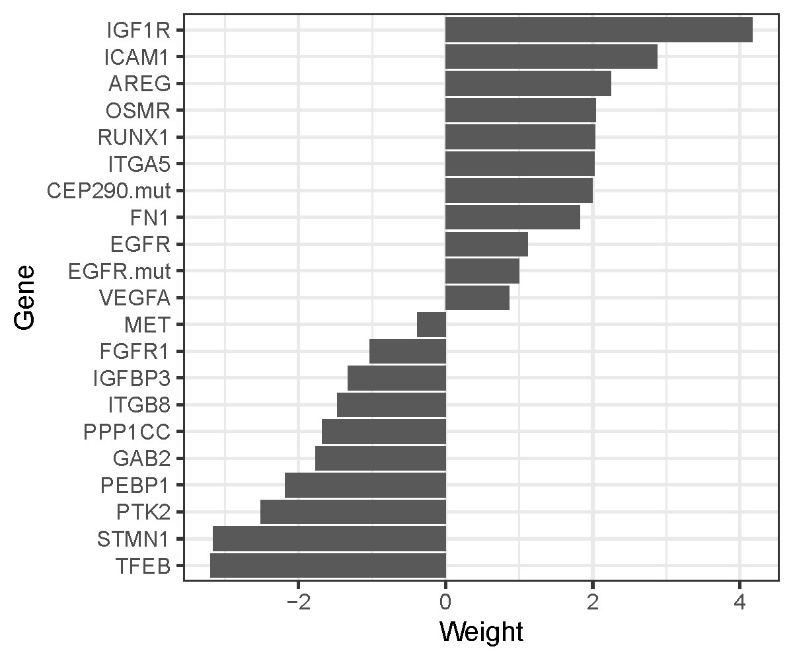


**Supplementary Figure 2. The expression levels and weight of predictor genes in LPS for training CDX models**

**A.** Boxplot of the expression level of each predictor genes in the unit of log_2_ TPM in the training samples, separated by response class. **B.** Bar plot of the weights of each predictor genes, ordered by decreasing value, in the LPS model. The abbreviations were: LPS, linear prediction score; CDX, cell line-derived xenograft; R: responsive models; PR, partial-responsive models; NR: non-responsive models; The abbreviations for the genes: *VEGFA,* Vascular Endothelial Growth Factor A gene*; EGFR,* Epidermal Growth Factor Receptor gene*; CEP290,* Centrosomal Protein 290 gene*; IGF1R,* Insulin-Like Growth Factor 1 Receptor gene*;* *OSMR,* oncostatin M receptor gene*; AREG,* Amphiregulin gene*; FN1,* Fibronectin 1 gene*; ITGA5,* Integrin Subunit Alpha 5 gene*; ICAM-1,* Intracellular adhesion molecule-1 gene*; RUNX1,* Runt-related transcription factor 1 gene; *FGFR1,* Fibroblast Growth Factor Receptor 1 gene*; PTK2,* nonreceptor protein tyrosine kinase 2 gene*; STMN1,* Stathmin 1 gene*; GAB2,* GRB2 Associated Binding Protein 2 gene*; ITGB8,* Integrin Subunit Beta 8 gene*; PEBP1,* Phosphatidylethanolamine-binding protein 1 gene*; PPP1CC,* serine/threonine-protein phosphatase PP1-gamma catalytic subunit gene*; TFEB,* Transcription factor EB gene*; IGFBP3,* Insulin Like Growth Factor Binding Protein 3 gene*; c-Met,* MET Proto-Oncogene gene.

Supplemental Figure 3


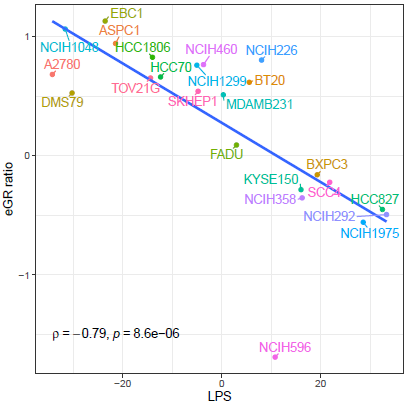


**Supplementary Figure 3. Correlation of eGR ratio with LPS values**

The correlation between the eGR ratio and LPS across 23 CDX models was tested. The calculations for these parameters were detailed in the method section. The x-axis represented the LPS values and y-axis represented the eGR ratio for each model. Each model was represented by a specific color for better separation. A significant inverse correlation was evident, with a Spearman correlation coefficient of -0.79 (*p* < 0.0001), indicating a significant negative association between the LPS value and efficacy endpoint. The abbreviations were: eGR, exponential growth rate; LPS, linear prediction score; CDX, cell line-derived xenograft

Supplementary Table 1

| **CDX Model** | **Cancer type** | **EGFR genetic status** | **c-Met genetic status** | **TGI (%)** | **Response to TAVO412** |
| --- | --- | --- | --- | --- | --- |
| **NCI-H596** | NSCLC | WT | WT | 98 | R |
| **FADU** | HNSCC | WT | WT | 95 | R |
| **HCC827** | NSCLC | Exon19 Del; AMP | WT | 94 | R |
| **NCIH1975** | NSCLC | Exon21 L858R; Exon 20 T790M | WT | 94 | R |
| **NCIH292** | NSCLC | WT | WT | 94 | R |
| **NCI-H358** | NSCLC | WT | WT | 89 | R |
| **KYSE150** | ESCC | WT | WT | 83 | R |
| **SCC4** | HNSCC | WT | WT | 82 | R |
| **BXPC3** | PDAC | WT | WT | 80 | R |
| **SKHEP1** | HCC | WT | WT | 66 | PR |
| **MDA-MB-231** | TNBC | WT | WT | 62 | PR |
| **DMS79** | SCLC | WT | WT | 58 | PR |
| **BT20** | TNBC | AMP | WT | 56 | PR |
| **A2780** | OC | WT | WT | 54 | PR |
| **NCIH226** | NSCLC | WT | WT | 46 | PR |
| **NCIH1299** | NSCLC | WT | WT | 43 | PR |
| **TOV21G** | OC | WT | WT | 38 | PR |
| **NCI-H460** | NSCLC | WT | WT | 36 | PR |
| **HCC1806** | TNBC | WT | WT | 28 | NR |
| **HCC70** | TNBC | WT | WT | 26 | NR |
| **ASPC1** | PDAC | WT | WT | -1 | NR |
| **NCI-H1048** | SCLC | WT | WT | -1 | NR |
| **EBC1** | NSCLC | WT | WT; AMP | -17 | NR |

**Supplementary Table 1: Summary of TGI values of TAVO412 across 23 CDX models**

The cancer types and genetic profiles of EGFR and c-Met (Cancer Cell Line Encyclopedia: <https://depmap.org/portal>) were summarized for each of the 23 CDX models. The TGI values and the corresponding response types to TAVO412 were also listed in the table. The TGI value was calculated with the data of the last observation day for each model. Based on the response to TAVO412, the models were categorized into 3 classes - R: responsive models (TGI ≥ 70%); PR, partial-responsive models (30% ≤ TGI < 70%); NR: non-responsive models (TGI < 30%). The abbreviations were: CDX, cell line-derived xenograft; TGI, tumor growth inhibition; NSCLC, non-small cell lung cancer; HNSCC, head and neck squamous cell carcinoma; ESCC, esophageal squamous cell carcinoma; HCC, hepatocellular carcinoma; SCLC, small-cell lung cancer; OC, ovarian cancer; PDAC, pancreatic ductal adenocarcinoma; TNBC, triple negative breast cancer; WT, wild type; AMP, amplification

Supplementary Table 2

| **PDX model** | **Cancer** | **EGFR genetic status** | **c-Met**  **genetic status** | **TAVO412**  **TGI (%)** | **Amivantamab**  **TGI (%)** | **Response to TAVO412** |
| --- | --- | --- | --- | --- | --- | --- |
| **LU2503** | NSCLC | WT | Exon-14 skipping;  Amplification | 99 | 100 | R |
| **LU-01-1377** | SCLC | Exon 21: L858R | Over-expression | 99 | 0 | R |
| **LU1901** | NSCLC | Exon18: G719A | Amplification | 85 | 35 | R |
| **LU-01-1623** | NSCLC | Exon19 Del | F569L | 84 | 53 | R |
| **LU-01-0506** | NSCLC | Exon18: E709A; G719A | Amplification | 83 | 37 | R |
| **LU5381** | NSCLC | WT | Exon-14 skipping;  Amplification | 75 | 62 | R |
| **LU-01-1649** | NSCLC | Exon 21: L858R | L1195V (Alias: L1213V) | 75 | 82 | R |
| **LDI-0015200717** | NSCLC | Exon19 Del,  Exon 20: T790M; C797S | WT | 51 | -3 | PR |
| **LU3075** | NSCLC | Exon-20 insertions  (P772_H773insDNP) | WT | 49 | 44 | PR |

**Supplementary Table 2: Summary of TGI values across 9 PDX models**

The cancer types and genetic profiles of EGFR and c-Met (provided by Crownbio and Wuxi through next-generation sequencing) were summarized for each of the 9 PDX models. The TGI values and the corresponding response types to TAVO412 were also listed in the table. The TGI value was calculated with the data of the last observation day for each model. Based on the response to TAVO412, the models were categorized into 2 classes - R: responsive models (TGI ≥ 70%); PR, partial-responsive models (30% ≤ TGI < 70%). The abbreviations were: PDX, patient-derived xenograft; TGI, tumor growth inhibition; NSCLC, non-small cell lung cancer; SCLC, small cell lung cancer; WT, wild type

Supplementary Table 3

| **Genes** | **Type** | **Association of Encoded Protein with Signal Pathways/Functions** | **References** |
| --- | --- | --- | --- |
| ***VEGFA*** | + | Circulating VEGF-A level is a predictive biomarker for bevacizumab in breast, pancreatic, and gastric cancers | 33 |
| ***EGFR*** | + | The expression, amplification and mutations (e.g., T790M) are predictive biomarkers for cetuximab and EGFR-TKIs | 20,34,35 |
| ***EGFR mutation*** | + | EGFR driver mutations are valuable biomarkers for predicting response to tyrosine kinase inhibitors (TKIs) in patients with metastatic lung adenocarcinoma | 35 |
| ***CEP290***  ***mutation*** | + | Involved in ciliary assembly and trafficking; CEP290 was expressed in hepatocellular carcinoma (HCC) tissues and various liver cancer cell lines, and play crucial roles in the proliferation, migration, infiltration, and ferroptosis of HCC cells. | 43 |
| ***IGF1R*** | + | Links to EGFR over-expression as commonly co-expressed; IGF signaling confers EGFR-TKI resistance due to the activation of MAPK and PI3K-AKT signaling pathways. | 36,37 |
| ***OSMR*** | + | It contributes to the regulation of local immune response and extracellular matrix processes in glioblastoma multiforme; OSMR is a co-receptor for EGFR, and has been shown to enhance EGFR signaling in glioblastoma | 38 |
| ***AREG*** | + | One of the major ligands of EGFR; EGFR activation has a longer half‐life stimulated by AREG than by EGF, and is either stabilized or recycled to the cell surface, resulting in overexpression of EGFR at the protein level | 39 |
| ***FN1*** | + | One of the key genes upregulated by the AREG-EGFR axis; high levels of FN1 mRNA and protein were observed in breast cancer cell lines that also express high levels of AREG | 40 |
| ***ITGA5*** | + | Encodes integrin subunit α5, which combined with ITGB1 to form Integrin α5β1, a receptor for FN1 | 40 |
| ***ICAM1*** | + | A downstream molecule of EGFR pathway, which is up-regulated by PI3K/Akt signaling through the activation of EGFR by the ligand AREG; ICAM1 overexpression is associated with tumor metastasis. | 41 |
| ***RUNX1*** | + | Promotes phosphorylated form of EGFR and upregulates EGFR at the transcriptional level by directly binding to the promoter of the EGFR gene | 42 |

**Supplementary Table 3: Functions of genes positively predicting the efficacy of TAVO412**

The genes that positively (+) predicted TAVO412 efficacy were listed. The association between the proteins encoded by these genes and the signal pathways/functions related to TAVO412 targets were summarized. For TAVO412 targets, the potential to be biomarkers was illustrated. The abbreviations for the genes: *VEGFA,* Vascular Endothelial Growth Factor A gene*; EGFR,* Epidermal Growth Factor Receptor gene*; CEP290,* Centrosomal Protein 290 gene*; IGF1R,* Insulin-Like Growth Factor 1 Receptor gene*;* *OSMR,* oncostatin M receptor gene*; AREG,* Amphiregulin gene*; FN1,* Fibronectin 1 gene*; ITGA5,* Integrin Subunit Alpha 5 gene*; ICAM-1,* Intracellular adhesion molecule-1 gene*; RUNX1,* Runt-related transcription factor 1 gene.

Supplementary Table 4

| **Genes** | **Type** | **Association of Encoded Protein with Signal Pathways/Functions** | **References** |
| --- | --- | --- | --- |
| ***FGFR1*** | - | Responsible for acquired resistance to afatinib and AZD9291; aberrant activations of bFGF/FGFR are also essential alternative angiogenic pathways that induce drug resistance to anti-VEGFR therapy | 35,44 |
| ***PTK2*** | - | The hyperphosphorylation of PTK2 occurs in various EGFR-TKI-resistant NSCLCs | 45 |
| ***STMN1*** | - | Encodes oncoprotein 18, which is a cytosolic phosphoprotein and a key regulator of cell division; overexpression of STMN1 confers EGFR-TKI resistance through the AKT/FOXM1/STMN1 pathway | 46 |
| ***GAB2*** | - | This scaffolding protein serves as a critical signaling amplifier downstream of tyrosine kinase receptors; GAB2 was shown to be one of the key proteins conferring EGFR-TKI resistance | 47 |
| ***ITGB8*** | - | ITGB8 encodes the β subunit of integrin (Integrin beta-8), which is upregulated in some types of cancer. Its overexpression was shown to be responsible for gefitinib resistance in hepatic cancer in a preclinical setting | 48 |
| ***PEBP1*** | - | Also know as Raf kinase inhibitor protein (RKIP), which is an endogenous inhibitor of Raf and it negatively regulates the Raf/MEK/ERK-signaling cascade. PEBP1 is a well-established metastasis suppressor, and the downregulation was associated with tumor progression | 49 |
| ***PPP1CC*** | - | PPP1CC gene encodes serine/threonine-protein phosphatase PP1-gamma catalytic subunit (PP1γ), which is aberrantly expressed in various cancers | 50 |
| ***MET*** | - | MET gene amplification and exon 14 skipping mutations are clinically recognized biomarkers for MET targeted therapies, whereas the clinical utility of c-Met overexpression as a biomarker has not been definitively established | 51 |
| ***TFEB*** | - | Encodes transcription factor EB, a master regulator of lysosomal function where EGFR proteolysis occurs. Hypoxia reduced mTORc1 activity and its ability to activate TFEB, thus preventing EGFR degradation. | 52 |
| ***IGFBP3*** | - | IGF binding protein 3 (IGFBP3) usually functions as a negative regulator of IGF1R signaling by binding to the ligands, so the downregulation of IGFBP3 is related to EGFR-TKI resistance, although not significant | 53,54 |

**Supplementary Table 4: Functions of genes negatively predicting the efficacy of TAVO412**

The genes that negatively (-) predicted TAVO412 efficacy were listed. The association between the proteins encoded by these genes and the signal pathways/functions related to TAVO412 targets were summarized if published reports are available, otherwise the major function of the genes in cancer progression were listed. For TAVO412 targets, the potential to be biomarkers was illustrated. The abbreviations were for the genes: *FGFR1,* Fibroblast Growth Factor Receptor 1 gene*; PTK2,* nonreceptor protein tyrosine kinase 2 gene*; STMN1,* Stathmin 1 gene*; GAB2,* GRB2 Associated Binding Protein 2 gene*; ITGB8,* Integrin Subunit Beta 8 gene*; PEBP1,* Phosphatidylethanolamine-binding protein 1 gene*; PPP1CC,* serine/threonine-protein phosphatase PP1-gamma catalytic subunit gene*; TFEB,* Transcription factor EB gene*; IGFBP3,* Insulin Like Growth Factor Binding Protein 3 gene*; c-Met,* MET Proto-Oncogene gene.
